# Analyzing Clustered Data: Why and How to Account for Multiple Observations Nested within a Study Participant?

**DOI:** 10.1371/journal.pone.0146721

**Published:** 2016-01-14

**Authors:** Erika L. Moen, Catherine J. Fricano-Kugler, Bryan W. Luikart, A. James O’Malley

**Affiliations:** 1 The Dartmouth Institute for Health Policy and Clinical Practice, Geisel School of Medicine at Dartmouth, Lebanon, New Hampshire, United States of America; 2 Department of Epidemiology, Geisel School of Medicine at Dartmouth, Lebanon, New Hampshire, United States of America; 3 Department of Physiology and Neurobiology, Geisel School of Medicine at Dartmouth, Lebanon, New Hampshire, United States of America; 4 Department of Biomedical Data Science, Geisel School of Medicine at Dartmouth, Lebanon, New Hampshire, United States of America; Indiana University, UNITED STATES

## Abstract

A conventional study design among medical and biological experimentalists involves collecting multiple measurements from a study subject. For example, experiments utilizing mouse models in neuroscience often involve collecting multiple neuron measurements per mouse to increase the number of observations without requiring a large number of mice. This leads to a form of statistical dependence referred to as clustering. Inappropriate analyses of clustered data have resulted in several recent critiques of neuroscience research that suggest the bar for statistical analyses within the field is set too low. We compare naïve analytical approaches to marginal, fixed-effect, and mixed-effect models and provide guidelines for when each of these models is most appropriate based on study design. We demonstrate the influence of clustering on a between-mouse treatment effect, a within-mouse treatment effect, and an interaction effect between the two. Our analyses demonstrate that these statistical approaches can give substantially different results, primarily when the analyses include a between-mouse treatment effect. In a novel analysis from a neuroscience perspective, we also refine the mixed-effect approach through the inclusion of an aggregate mouse-level counterpart to a within-mouse (neuron level) treatment as an additional predictor by adapting an advanced modeling technique that has been used in social science research and show that this yields more informative results. Based on these findings, we emphasize the importance of appropriate analyses of clustered data, and we aim for this work to serve as a resource for when one is deciding which approach will work best for a given study.

## Introduction

The reproducibility of scientific findings relies on using statistical approaches that reflect the design through which data are obtained in the study. The nature of statistical research design within the field of neuroscience has recently become a focus of criticism by some groups who suggest that a higher bar needs to be set for statistical analyses in published work, especially concerning those experiments that contain clustered data [1–4]. Clustered data can occur when there are multiple measurements of the same subject (e.g., due to making repeated measurements over time, space, or simply pure replicate measurements) and are common in many areas of experimental medicine and biology. Observations from the same subject tend to be correlated, meaning that not all observations in the study are independent, and the total sample-size is not a true reflection of the information/level-of-evidence in the data.

A common study design in neuroscience experiments with murine models is to analyze an effect at the level of individual neurons, sampling multiple neurons per mouse. A neuroscientist performing experiments with very few mice will lean towards this approach, as they want to maximize the sample-size of their study. Inappropriate statistical analyses that are common with these data occur when the correlation of neurons from the same mouse is ignored and each neuron is treated as an independent observation. A previously published analysis of the August 2008 issue of *Nature Neuroscience* found that the overwhelming majority of papers (17 out of 19) analyzed clustered data with replicates that were not statistically independent [2]. However, most of those papers (82%) did not have sufficient information for the reader to determine whether each observation was considered independent in the analyses [2]. Another more recent review of the literature found that 53% of 314 reviewed studies from five high-level neuroscience journals did not correctly account for the clustered structure of their data in the analyses [1]. Such concerns are not restricted to neuroscience; for example, researchers analyzing clinical trials are also urged to consider patients clustered with physicians in their analytical approaches to account for variation between physicians [5].

In this work, we explore data from a study published by members of our group to demonstrate several different approaches for analyzing clustered data [6]. We aim to provide an understanding of the pros and cons and the appropriate interpretation of results under each method. These approaches and the insights we provide can be applied to any study design that contains clustered data.

## Materials and Methods

### Experimental design of illustrative neuroscience experiment

The study that generated the data we use in this paper examined the effects of Pten knockdown and fatty acid delivery on soma size of neurons in the brain [6]. Pten knockdown was measured at the level of individual neurons and varied within mice, and fatty acid exposure was randomized at the level of the mouse and thus varied between mice. Briefly, to investigate the effect of Pten knockdown on soma size, mice were co-injected with an FUGW-based lentivirus expressing both GFP and a shRNA targeting the *Pten* coding region and a control virus expressing only mCherry. The number of neurons in the GFP Pten knockdown and control mCherry groups varied within and between mice depending on the level of infection of each virus. When a study is designed in this way, each mouse can serve as its own control because the shRNA for the target of interest is not meant to infect every neuron. The mice were also randomized into three groups that either received a mixture of fatty acids, vehicle control, or remained naïve to treatment. To determine whether the fatty acid environment of the brain influences soma size, osmotic pumps were implanted subcutaneously to deliver either a constant mixture of palmitic, myristic, and palmitoleic acids or vehicle control.

**Fig 1:**
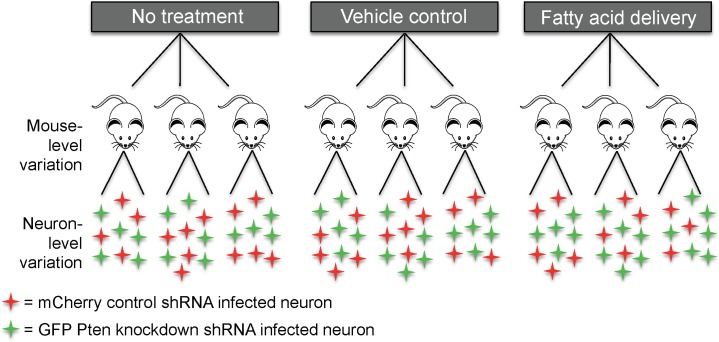
Experimental design underlying the neuroscience dataset. The mouse-level treatment was fatty acid delivery, vehicle control, or no treatment. The neuron-level treatment was Pten or control shRNA. The two levels of treatment resulted in a hierarchical study design with a between-mouse and within-mouse treatment factor.

The original study aimed to investigate the existence of an interaction between the within-mouse factor (Pten knockdown) and between-mouse factor (fatty acid exposure). Their hypotheses were (i) that the effect of Pten knockdown would be enhanced under fatty acid delivery relative to vehicle, and (ii) that Pten knockdown would have a larger impact under fatty acid delivery compared with shRNA control. Downstream analyses thus had layers of hierarchy, extending from neurons per mouse to mice per fatty acid exposure group (Fig 1). There are several research questions one could ask with the data generated from this experiment, including those not considered in the original study (Table 1). The subsets of mice that would comprise a dedicated study of each such question are indicated in Table 1 and analyzed herein as hypothetical distinct experiments.

**Table 1:** Potential research questions testable by Pten knockdown and fatty acid environment study data.

Research question	Relevant neuron/mouse population in a dedicated study of the research question
1. Is there an effect of fatty acid on soma size?	Neurons not exposed to Pten shRNA in mice exposed to fatty acid or vehicle control
2. Is there an effect of Pten knockdown on soma size?	Mice not exposed to fatty acid or vehicle control
3. Does the proportion of neurons with Pten shRNA ($\bar{\text{Pten}}$) affect soma size?	Mice not exposed to fatty acid or vehicle control
4. Is there an interaction effect of Pten knockdown and fatty acid on soma size?	Mice exposed to fatty acid or vehicle control

Note: These examples are expanded upon in later sections of the text.

### Naïve statistical models

All analyses were performed in Stata 13.1 [7]. For the Stata code used to perform the statistical models, please see https://github.com/elmoen/clustered_data_code.git. The pertinent commands for these analyses in Matlab, R, and SAS are also included.

There are two common linear regression approaches to analyzing clustered data that in general do not properly account for clustering. The first is a regression that includes each neuron measurement as an independent observation, referred to as the “complete-pooling” approach. In the case of a linear regression, this approach corresponds to estimating the model:

$$Y_{ij}=\beta_{0}+\beta_{1}X_{ij}+\varepsilon_{ij}$$ (1)

where *Y*_*ij*_ is the dependent variable representing observation *j* in subject *i*, *X*_*ij*_ is a corresponding covariate, and *β*_0_ and *β*_1_ are unknown regression coefficients representing the intercept parameter and the slope coefficients of the covariates, respectively. The error, *ε*_*ij*_, is often assumed to have a normal distribution around a mean of 0 and a constant variance *σ*^2^ among all observations:

$$\varepsilon_{ij}∼\text{Normal}\left(0,\sigma^{2}\right)$$ (2)

Crucially, the model assumes independence of all observations regardless of which subject they are clustered within.

The second naïve approach is to reduce the observations to the number of subjects in the study by using a summary measure (e.g., mean). This is referred to as an aggregate or ecological model and is depicted below:

$${\bar{Y}}_{i}=\beta_{0}{\bar{X}}_{i}+{\bar{\varepsilon }}_{i}$$ (3)

where ${\bar{\varepsilon }}_{i}∼\text{Normal}\left(0,{\bar{\sigma }}^{2}\right)$. In its basic form, this approach does not take into account the extent to which the precision of the sample mean varies between mice. Weighting by the number of observations for each subject can help to account for the varying precision of the repeated measurements for each subject. The error term including analytic weights is depicted as ${\bar{\varepsilon }}_{i}∼\text{Normal}\left(0,\sigma^{2}w_{i}\right)$, where *w*_*i*_ = 1/*n*_*i*_. Although the above two approaches are frequently implemented, as we will demonstrate in our study, they fail to accurately embody the study design even when including analytic weights and therefore can produce misleading results.

### Statistical models for handling clustered data

The three primary categories of approaches that are appropriate for clustered data we will discuss are marginal models, fixed-effect models, and mixed-effect models.

#### Marginal model

The first approach we consider is identical in its modeling of the mean to the linear regression represented in Eq 1 but differs in its assumptions about the statistical dependence of observations from the same subject. Because the expected value of the outcomes *Y*_*ij*_ conditional on the predictors *X*_*ij*_, given by

$$E\left[Y_{ij}|X_{ij}\right]=\beta_{0}+\beta_{1}X_{ij},$$ (4)

depends on no subject-specific parameters (these would have the subscript *i*), the model for the mean is often referred to as a population-averaged or “marginal regression model”. Marginal models only make assumptions about the relationship of the expected value of the outcome to the predictors in the model, and do not require the conditional distribution of the observations given the covariates to satisfy a particular distribution for the model to hold. This approach has led to a method of estimation known as generalized estimating equations (GEE) that adjusts standard regression estimators for clustering as opposed to relying on a fully-specified model for estimation. The GEE estimation procedure for (4) assumes E[*ε*_*ij*_] = 0 and typically that var[*ε*_*ij*_] = *σ*^2^ but does not assume a particular distribution for *ε*_*ij*_ nor does it rely on the correct specification of the correlation structure between the observations on the same subject, making it a robust procedure. Specifically, the correlation structure specified with the GEE procedure can be viewed as a tuning parameter, and an estimator will perform better with small sample sizes if it coheres with the true correlation structure. For example, an exchangeable correlation structure with constant variance would specify that each measure from the subject has the same variance and all pairs of measurements from the subject are equally correlated. The crucial point of marginal models is that the model does not have to be specified correctly in order for the confidence intervals and p-values obtained from GEE to be appropriately calibrated; the penalty for incorrect specification is restricted to the precision, or standard error, of the results [8].

#### Fixed-effect model

Fixed-effect models differ from marginal regression models through their inclusion of subject-specific regression coefficients. A fixed-effect regression model includes the indicator variables ${\left\{ind_{ik}\right\}}_{k=1}^{K}$, where *ind*_*ik*_ = 1 if *i* = *k* (the k’th subject) and 0 otherwise (*K* denotes the number of subjects), as additional covariates, yielding the model:

$$Y_{ij}=\beta_{0}+\beta_{1}ind_{i1}+\cdots +\beta_{K}ind_{iK}+\beta_{K+1}X_{ij}+\varepsilon_{ij}$$ (5)

where *β*_0_ is a dedicated intercept and ${\left\{\beta_{k}\right\}}_{k=1}^{K}$ denote the subject fixed effects with the constraint *β*_*K*_ = 0 so that the effects of other subjects represent differences in the expected outcome with respect to that of individual *K* (the “baseline” subject). The regression coefficient of *X*_*ij*_, which is of primary interest herein, is now *β*_*K+1*_. The interpretation is as for the marginal model in that *β*_*K+1*_ represents the effect of a 1-unit change in *X*_*ij*_ on the expected value of *Y*_*ij*_ given *X*_*ij*_, holding all other terms fixed. The error, *ε*_*ij*_, is often assumed to have a normal distribution around a mean of 0 and a constant variance *σ*^2^ among observations from the same subject:

$$\varepsilon_{ij}∼\text{Normal}\left(0,\sigma^{2}\right)$$ (6)

If individual variation is treated as a fixed effect (i.e., repeated experiments would involve the same subjects), a strict interpretation of inferences under a fixed-effect approach is that they are applicable only to those subjects who were in the experiment that was performed. Fixed-effect models are not used to model the relationship between an outcome and an explanatory variable that only varies between subjects because of the perfect collinearity, or exact linear relationship, between the explanatory variable and the subject fixed effects. In other words, after including fixed effects for subjects, the estimated model perfectly interpolates the subject means leaving no information to determine the effect of a subject-level explanatory variable; the individual effect of each subject is confounded with the effect of the subject-level explanatory variable.

#### Mixed-effect model

A mixed-effect model is also subject-specific, but the individual subjects are represented as random draws from a hypothetical population, and their coefficients are characterized by a specified probability distribution. This model is preferred philosophically for instances where the researcher is interested in inferring results for the population of individuals, not just those who participated in the experiment. Specifically, the mixed-effect model includes the individual subject as a random effect and is represented by the following formula:

$$Y_{ij}=\beta_{0}+\beta_{1}X_{ij}+\theta_{i}+\varepsilon_{ij}$$ (7)

where *ε*_*ij*_ ∼ Normal(0, *σ*^2^), the intercept *β*_0_ is interpreted as an adjusted population mean, and *θ*_*i*_ denotes the random-effect of the subject labeled *i*, typically assumed to have a normal distribution around a mean of 0 and a constant variance τ^2^:

$$\theta_{i}∼\text{Normal}\left(0,\tau^{2}\right)$$ (8)

Random effects essentially give structure to the error within the experiment, and refer to effects that can differ among subjects [9] while *β*_1_ in (7) is confusingly referred to as a “fixed effect” (distinct from fixed-effect model) because it does not vary across subjects and would not be subject to change if the experiment were repeated.

The mixed-effect model lets the data decide how much an estimate of a between-subject effect resembles the corresponding complete pooling model estimate versus the fixed-effect model estimate. Specifying robust standard errors makes the estimate robust to model misspecification due to incorrect variance function, the within-subject correlation structure, or distributional assumption. The difference between classical standard errors and robust standard errors can also be informative as to the extent of the misspecification of the model, and if the difference is large the data may warrant further scrutiny to determine whether a better model can be specified [10].

Random effect models rely on the assumption that the random effects are independent of the observed covariates *X* and the error terms ε for unbiased estimation of the regression parameters [11], while inferences (e.g., confidence intervals and *p*-values) depend additionally on the correctness of the random effects distribution assumption [12,13]. Under mixed-effects models the commonly used R-square measure of model fit is not as naturally defined as for standard linear regression models due to the presence of an observation-level and a subject-level in the same model. As a result, many procedures in statistical software packages, including the Stata xtmixed procedure used in this work, do not report R-square measures for models including random effects, and sometimes not even for marginal models. Because the variation in the data must be partitioned between the observation and subject levels, adding a predictor that improves the fit at the observation level may reveal more variability and possibly lower R-square at the subject level. Therefore, the traditional property of R-square as always increasing when more predictors are added to a model does not necessarily hold for mixed-effect models, which is why we believe there is a tendency to avoid reporting diagnostics such as R-square among software packages.

In the following sections, we discuss the above statistical approaches from a practical perspective using data from a recently completed neuroscience experiment (Table 2). We demonstrate the extent to which accounting for the between-mouse variation in the model affects the results of a between-mouse treatment effect, within-mouse treatment effect, and an interaction between these two effects.

**Table 2:** Characteristics of marginal, fixed-effect, and mixed-effect models.

Characteristic	Marginal	Fixed-effect	Mixed-effect
Distinguishes observations belonging to the same or different subjects	Yes^a^	Yes	Yes
Reliant on distribution of subject-specific effects	No	No	Yes
Subjects considered a sample from a population larger than the sample itself	Yes^a^	No	Yes
Computation handles few subjects well	No	Yes	No
Computation handles a very large number of subjects well	Yes	No	Yes
Noisy for few observations per subject	No	Yes	No
Computation handles a large number of observations per subject	Depends^b^	Yes	Yes
Accommodates variable observations per subject	Yes	Yes	Yes

Note: ^a^Only for calculation of standard errors.

^b^Problems can arise under some specifications of the working covariance structure and depending on the estimation method used.

## Results

### Effect of between-mouse treatment

**Fig 2:**
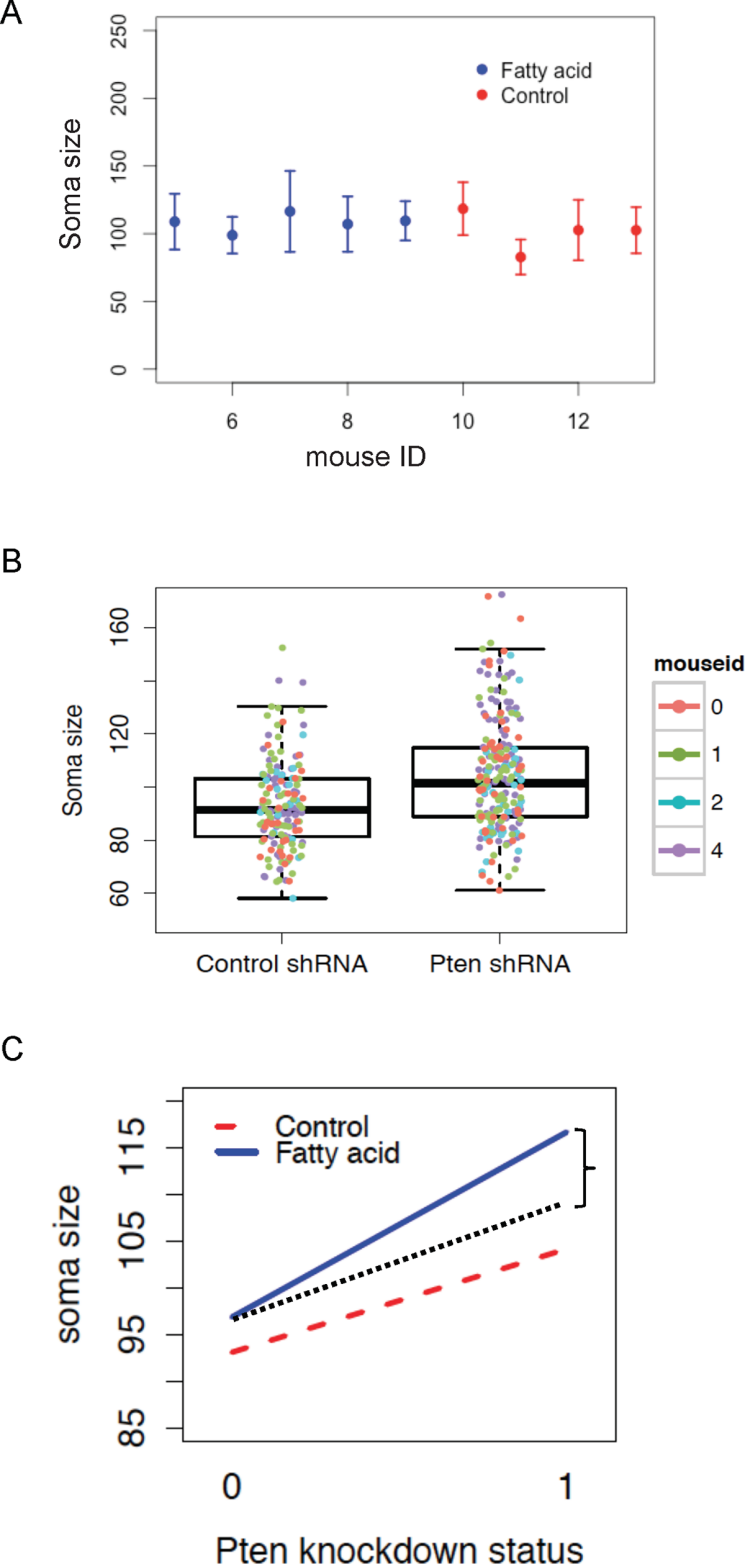
Visualization of clustered data. (A) Visualization of a between-mouse factor. Each point represents the mean soma size of a mouse ± standard error (SE). [SE = standard deviation (mean soma size)]. (B) Visualization of a within-mouse factor. Each point represents the soma size of an individual neuron within a mouse. The colors correspond to the mouse to which the neurons belonged. Each mouse has neurons with control and Pten shRNA. (C) Visualization of an interaction effect between the within-mouse and between-mouse factor. The red dotted line represents the vehicle control mice and the blue solid line represents the fatty acid delivery mice. Pten knockdown status 0 = control shRNA and 1 = Pten shRNA. The black dotted line depicts the expected result if there were no interaction effect, and the space between the black dotted line and the blue line, denoted by the curly bracket, represents the size of the interaction effect.

One experimental design that a researcher may propose is to analyze the effect of an exposure or treatment on a neuronal phenotype. To do so, the researcher may have a group of mice that received a vehicle control, and another group(s) of mice that received the treatment(s). We describe this type of experiment as having a “between-mouse” treatment effect, because the treatment only varies between mice (Fig 2A).

#### Neuron-level analysis: pooled linear regression

The first research question in Table 1 is investigating the effect of fatty acid delivery compared with vehicle control on soma size. A common approach to answer this question may be to perform a simple linear regression including all observations as independent:

$${\text{somasize}}_{ij}=\beta_{0}+\beta_{1}{\text{fattyacid}}_{i}+\varepsilon_{ij}$$

where *ε*_*ij*_ ∼ Normal(0, *σ*^2^), *i* denotes the mouse, *j* denotes the neuron within the mouse. (To emphasize the study design and thereby the structure of the data, the fattyacid variable only includes the subscript *i*.) When we regress soma size on fatty acid exposure, we obtain an estimated coefficient of 3.15, meaning the model is estimating that the mice in the fatty acid group have soma sizes that are 3.15 units larger on average than the mice in the vehicle control group, and the difference only reaches borderline significance (*p* = 0.099, Table 3). The complete-pooling approach does not handle the issue of non-independence generated from multiple neurons per mouse. This fallacy is exemplified by the fact that sample-size quantified as the total number of neurons is increased by either adding more neurons per mouse or increasing the number of neurons by adding more mice, but these are not equivalent alterations to the study design. Increasing the number of neurons per mouse without accounting for clustering artificially inflates the precision of inferences on between mouse effects. This can make it easier to detect statistically significant results for a mouse-level factor as the extent to which estimated effects can vary by chance is underestimated.

**Table 3:** Results of fatty acid exposure on soma size from different regression models (Research Q1).

	Coefficient	Std Err	*p*-value	95% CI
Neuron-level linear regression	3.15	1.90	0.099	-0.60, 6.90
Mouse-level regression (mean); no weighting	0.61	6.37	0.926	-14.47, 15.69
Mouse-level regression (mean); analytic weights	3.15	4.73	0.527	-8.04, 14.33
Marginal regression^a^	2.31	4.86	0.635	-7.22, 11.83
Fixed effect regression^a^	n/a			
Mixed-effect regression^a^	1.56	5.04	0.756	-8.32, 11.44

Note: The coefficient captures the effect of the fatty acid environment compared to the vehicle control.

^a^These are neuron-level regressions.

The inappropriate use of complete-pooling analyses is the primary motivator for the scrutiny being applied to the statistical research design of neuroscience experiments involving clustered data [1,2]. The only instance when a Student’s *t*-test or complete-pooling model is appropriate would be when the variation of the observations is only due to unobserved factors between neurons, not unobserved factors between mice. In other words, if two neurons from the same mouse are not any more correlated than two neurons from two separate mice, then each neuron can be considered an independent observation.

#### Mouse-level analysis: simple regression with mean soma size per mouse

A mouse-level approach that removes the clustering completely would be to use the mean soma size ($\bar{\text{somasize}}$) for each mouse as the outcome in the regression model. The varying precision of the measurements on different mice can be accounted by weighting by the number of observations (*w*_*i*_) made on the mouse:

$${\bar{\text{somasize}}}_{i}=\beta_{0}+\beta_{1}{\text{fattyacid}}_{i}+{\bar{\varepsilon }}_{i}$$

where, for the model including analytic weights, ${\bar{\varepsilon }}_{i}∼\text{Normal}\left(0,\sigma^{2}w_{i}\right)$ and *w*_*i*_ = 1/*n*_*i*_.

When we perform the mouse-level linear regression without weighting, we observe there is no significant difference of mean soma size between mice in the fatty acid exposure group compared with the vehicle control group (coefficient = 0.61, *p* = 0.926) (Table 3), reflecting the severe under-stating of the information in the data from ignoring the variability in the number of neurons per mouse. The regression coefficient of the weighted model is the same as the naïve (complete-pooling) regression model, 3.15, but the standard error is now 4.73 and *p* = 0.527. The standard error of the coefficient is different from that for the naïve model because the variance of the error term is estimated only using variation between mice. However, reducing the data to a mean per mouse still results in a loss of information, even after weighting, because the observed variability between neurons within each mouse is ignored (Table 3).

#### Marginal, fixed-effect, and mixed-effect models

Thus far, one approach considers the sample-size equal to the total number of neurons and the other reduces the data to one measurement for each mouse so that the sample-size equals the number of mice. With clustered data, the extent of the variation within and between subjects influences the effective sample size of the study, such that it actually lies somewhere between the number of subjects (mice) and the total number of observations (neurons). The comparison of variances is known as the intraclass correlation coefficient (ICC) [14], which compares the variance between subjects (*τ*^2^) to the variance within subjects (*σ*^2^), and is calculated with the following equation:

$$\text{ICC}=\tau^{2}/\left(\tau^{2}+\sigma^{2}\right)$$ (9)

We demonstrate how the ICC can be used to calculate the effective sample size of a study in S1 Appendix. The ICC can range from 0 (no correlation) to 1 (perfect correlation), and in this experiment the ICC of neurons within a mouse equals 0.2. Thus, the linear regression models that include all neurons while accounting for between-mouse lie between the complete-pooling and no-pooling approaches in terms of effective sample size quantification (S1 Appendix). The most basic of these is the model that appears identical to the complete-pooling approach in terms of its modeling of the mean but deviates in the statistical independence assumptions of neurons from the same mouse. This marginal regression model (Eq 4) is given by:

$${\text{somasize}}_{ij}=\beta_{0}+\beta_{1}{\text{fattyacid}}_{i}+\varepsilon_{ij},$$

where E[*ε*_*ij*_] = 0 and *var*[*ε*_*ij*_] = *σ*^2^. We now observe that the soma sizes of mice in the fatty acid exposure group are far from being significantly different from those in the vehicle control group (*p* = 0.635, Table 3). The estimate of the coefficient calculated with marginal regression (2.31) is between the estimate calculated with the neuron-level regression and the mouse-level regression without weighting, demonstrating the overestimation of the estimate in the neuron-level regression model and underestimation in the mouse-level regression without weighting.

For this experimental design, a fixed-effect model is not appropriate because of the perfect collinearity between mouse-level variation and fatty acid treatment assignment (both vary only at the level of the mouse and so we cannot separate the unique effect of each mouse from the effect of the environment). Most statistical programs would likely give an error or warning message if we attempted to include every mouse identifier as a fixed-effect to alert the user of the occurrence of perfect collinearity between the predictors and might report a missing value indicator for the regression coefficient.

However, we can use a mixed-effect model regressing soma size on fatty acid exposure with individual mouse as a random effect, denoted by *θ*_*i*_ (Eq 7):

$${\text{somasize}}_{ij}=\beta_{0}+\beta_{1}{\text{fattyacid}}_{i}+\theta_{i}+\varepsilon_{ij},$$

where *θ*_*i*_ ∼ Normal(0, *τ*^2^) and *ε*_*ij*_ ∼ Normal(0, *σ*^2^). The mixed-effect model uses partial-pooling, so the overall fixed effect for the fatty acid environment is estimated while allowing the intercept to vary with mouse as a random effect. The use of robust variance estimation in the model would ensure the legitimacy of the standard errors (thus also confidence intervals and *p*-values) is not fully reliant on the correct specification of the model (see https://github.com/elmoen/clustered_data_code for Stata code). When we perform the mixed-effect model the estimate of the regression coefficient (1.56) has a wider confidence interval than the marginal regression model and is not significant (*p* = 0.756, Table 3).

In this experiment, the pooled linear regression underestimated the variation of the effect (std err = 1.90) and brought to *p*-value to a borderline significant level (*p* = 0.099 compared with *p*>0.5 for all other models, Table 3) The lower *p*-value obtained from the pooled regression may bias researchers towards thinking an association may exist, when the other models indicate clearly that there is none. Overall, it is clear that the choice of statistical approach has implications for the results in this example of a between-mouse treatment effect.

### Effect of within-mouse treatment

A within-mouse treatment effect occurs when the researcher designs an experiment where each mouse has measurements of the phenotype from both the control and treatment arm. We describe these experiments as having a “within-mouse” treatment effect, because the treatment varies at the level of neurons within mice.

#### Neuron-level analyses: Student’s *t*-test and pooled linear regression

The second research question in Table 1 is regarding how Pten knockdown affects soma size of individual neurons. To answer this, a researcher would want to compare the soma sizes of GFP Pten knockdown neurons with the control mCherry neurons in mice that were not exposed to any environmental treatment (Fig 2B). Because this study has two groups, a common approach is to use a two-sample Student’s *t*-test, with which we find a significant difference in soma size of Pten knockdown neurons compared with control neurons (*p*<0.001). If the number of neurons varied significantly between GFP Pten knockdown and mCherry control groups, a weighted two-sample Student’s *t-*test could be used to account for the differences in sample-size. Another similar naïve approach would be a complete-pooling approach to estimate the simple linear regression model:

$${\text{somasize}}_{ij}=\beta_{0}+\beta_{1}{\text{Pten}}_{ij}+\varepsilon_{ij}$$

where *ε*_*ij*_ ∼ Normal(0, *σ*^2^). When we perform this neuron-level regression, we observe a significant correlation between Pten knockdown and soma size (*p*<0.001, Table 4). The regression coefficient is 11.04, meaning that neurons with Pten knockdown have an estimated average increase in soma size of 11.04 units. However, the clustered structure of the data is ignored in these approaches.

**Table 4:** Results of Pten knockdown effect on soma size from different regression models (Research Q2).

	Coefficient	Std Err	*p*-value	95% CI
Neuron-level linear regression	11.04	1.98	<0.001	7.15, 14.92
Marginal regression	11.51	2.46	<0.001	6.70, 16.32
Fixed-effect regression	11.54	1.86	<0.001	7.88, 15.20
Mixed-effect regression	11.50	2.45	<0.001	6.70, 16.30
Mixed effect regression with $\bar{\text{Pten}}$ as an additional predictor	11.54	2.49	<0.001	6.66, 16.41

Note: The coefficient represents the effect of Pten knockdown on soma size.

#### Marginal, fixed-effect, and mixed-effect models

Next, we will perform the three regression models that directly account for clustering. The marginal regression to analyze the within-mouse treatment effect is given by:

$${\text{somasize}}_{ij}=\beta_{0}+\beta_{1}{\text{Pten}}_{ij}+\varepsilon_{ij}$$

where E[*ε*_*ij*_] = 0 and *var*[*ε*_*ij*_] = *σ*^2^. With the marginal model, we observe a significant effect of Pten knockdown on soma size (*p*<0.001). The regression coefficient represents an estimated increase of 11.51 soma size units due to Pten knockdown, which is greater than what we saw for the simple linear regression that did not take clustering into account (Table 4). Therefore, accounting for between-mouse variation increased the observable soma size differences due to Pten knockdown compared with the complete-pooling linear regression.

A fixed-effect linear regression could also be used when analyzing a within-mouse factor by including the set of dummy variables indicating mouse identity *mouse*_*ik*_ = 1 if *i = k* and 0 otherwise for *k* = 1, …, *K* in the model:

$${\text{somasize}}_{ij}=\beta_{0}+\beta_{1}mouse_{i1}+\cdots +\beta_{K}mouse_{iK}+\beta_{K+1}{\text{Pten}}_{ij}+\varepsilon_{ij}$$

where *ε*_*ij*_ ∼ Normal(0, *σ*^2^). This method accounts for between-mouse variability without making any assumptions about the mouse-level effects. When we estimate the fixed-effect model, we find the coefficient for the effect of Pten equals 11.54 with *p*<0.001 (Table 4).

The final linear regression model we will discuss for determining whether Pten knockdown affects soma size is a mixed-effect model with individual mouse as a random effect:

$${\text{somasize}}_{ij}=\beta_{0}+\beta_{1}{\text{Pten}}_{ij}+\theta_{i}+\varepsilon_{ij}$$

where *θ*_*i*_ ∼ Normal(0, *τ*^2^) and *ε*_*ij*_ ∼ Normal(0, *σ*^2^). Thus, the random effect *θ*_*i*_ represents the effect of the *i*th sampled mouse, whereas *β*_*i*_ in the fixed-effect model is the effect of mouse *i* itself and no assumption is imposed on its distribution. The results from the mixed-effect model most closely resemble the marginal model, with a coefficient of 11.50 and *p*<0.001 (Table 4).

Overall, the three regression models that account for the clustered structure of the data yield very similar results for the within-mouse treatment effect, and either approach would be valid. Accounting for clustering essentially eliminates the confounding due to between-mouse variation by adjusting the mean of all mice to be equal, which is why the effect of Pten knockdown is larger in these methods compared with the complete-pooling approach.

#### Effect of the proportion of neurons with Pten knockdown ($\bar{\text{Pten}}$) on soma size

The third research question in Table 1 is investigating the effect of an aggregate measure of Pten knockdown per mouse ($\bar{\text{Pten}}$) on soma size. We could hypothesize that any relationship of Pten knockdown to soma size would be captured by the relationship of the proportion of neurons with Pten knockdown per mouse to mean soma size per mouse. Thus, we would be analyzing the aggregate measure (proportion of neurons with Pten knockdown) and its relationship to mean soma size of the mouse:

$$\bar{{\text{somasize}}_{i}}=\beta_{0}+\beta_{1}{\bar{\text{Pten}}}_{i}+\bar{\varepsilon_{i}}$$

This is similar to ecological regression, a statistical technique used to calculate group behavior from aggregate data often used in the social sciences [15]. Although analyzing aggregate data may seem intuitive, especially to researchers that usually analyze data with one measurement per subject, it may incur a subtle bias due to the fact that the relationship at the aggregate (mouse) level need not be the same as at the observation (neuron) level, leading to a fallacy known as ecological bias. When we perform the simple mouse-level regression, using weighting to account for the varying number of neurons per mouse, we in fact observe a negative coefficient (-0.24) (Table 5). This negative coefficient demonstrates the ecological bias phenomenon, whereby the effect of an aggregate variable ($\bar{\text{Pten}}$) can be opposite to that of an individual variable (Pten).

**Table 5:** Results of ($\bar{\text{Pten}}$) knockdown effect on soma size from different regression models (Research Q3).

	Coefficient	Std Err	*p*-value	95% CI
Mouse-level regression ($\bar{\text{Pten}}$); weighting	-0.24	0.67	0.744	-2.36, 1.88
Marginal regression	-0.11	0.20	0.581	-0.50, 0.28
Mixed-effect regression	-0.004	0.19	0.981	-0.37, 0.37
Mixed-effect regression with Pten as an additional predictor	-0.11	0.20	0.562	-0.51, 0.28

Note: The coefficient represents the effect of $\bar{\text{Pten}}$ on soma size.

Next, we applied the marginal model with $\bar{\text{Pten}}$ as the independent variable and observe a negative coefficient (-0.11) that is not significant (*p* = 0.581). A fixed-effect model is not appropriate due to the perfect collinearity between the value of $\bar{\text{Pten}}$ and the mouse indicator. A mixed-effect model can be used, and we show results for the model with and without Pten knockdown status of individual neurons included as a fixed-effect (Table 5). The following model depicts the inclusion of both the aggregate and neuron-level measure of Pten knockdown as distinct predictors in the mixed-effect model:

$${\text{somasize}}_{ij}=\beta_{0}+\beta_{1}{\bar{\text{Pten}}}_{i}+\beta_{2}{\text{Pten}}_{ij}+\theta_{i}+\varepsilon_{ij}$$

It is interesting to note that when Pten knockdown status of each neuron is a predictor, the coefficient of $\bar{\text{Pten}}$ in the mixed effect model is the same as for the marginal model (-0.11) (Table 5). Furthermore, the effect of Pten knockdown estimated under this model (11.54) nearly equals that for the fixed effect model presented in the third and fifth rows of Table 4. The latter result reflects the fact that when $\bar{\text{Pten}}$ is not accounted for in the mixed effect model, both within and between-mouse variation is used to estimate the effect of Pten knockdown with the resulting estimate being a weighted average of the Pten and $\bar{\text{Pten}}$ effects in the combined model. Conversely, the effect of $\bar{\text{Pten}}$ when it alone is included in the mixed effect model is impacted by the (omitted) Pten variable because there are a greater number of observations on mice with larger values of $\bar{\text{Pten}}$ than on those with smaller values. Therefore, inclusion of the aggregate and individual predictors led to greater uniformity across the model estimates. One could also include $\bar{\text{Pten}}$ and Pten predictors in a marginal model as well, but not a fixed-effect model for the reasons of collinearity mentioned above.

In addition to accounting for the confounding of individual Pten by an aggregate $\bar{\text{Pten}}$, an advantage of estimating the mixed effect model with both effects is that it can provide valuable scientific insight. For example, here we observe that neurons infected with the Pten shRNA in a mouse with a smaller proportion of infected neurons (smaller $\bar{\text{Pten}}$) had a larger mean soma size relative to the unaffected neurons of the same mouse compared with the difference in mean soma size across infected and unaffected neurons in a mouse with a higher $\bar{\text{Pten}}$ infection. We could hypothesize that this is evidence of a biological phenomenon whereby there is less competition for space to grow when fewer neurons were infected with Pten shRNA.

#### Effect of the interaction between the within-mouse factor and between-mouse factor

An important aspect to this experimental design of the neuroscience study analyzed herein is that one can study the effect of Pten knockdown within a fatty acid environment compared with a control environment. While most studies traditionally focus on a single genetic or environmental variable, it is becoming increasingly clear that gene/gene and gene/environment interactions are important for the development of disease. This will necessitate more studies in the future needing to account for such interactions. To investigate an interaction effect, it is not enough to compare the significance levels of the soma size increase in Pten knockdown neurons between the environments, because the difference in soma size experienced by these two groups with Pten knockdown may not be significant. Therefore, it is important to state the significance of the difference, not differences in significance [4].

We performed a marginal, fixed-effect, and mixed-effect model to determine the significance of the interaction effect term between Pten knockdown and fatty acid delivery on soma size. The interaction term behaves more like a between-subject treatment effect with respect to clustering. We observe a significant effect of the interaction term using each model (Table 6). In this experiment, the interaction effect under the marginal model with an exchangeable working correlation structure has the highest estimated effect and standard error, but this is an idiosyncratic feature of these data, and it will not always be the case that marginal models with an exchangeable working correlation structure will lead to larger interaction effects.

**Table 6:** Results of interaction between Pten knockdown and fatty acid exposure models (Research Q4).

	Coefficient	Std Err	*p*-value	95% CI
Marginal regression	10.38	5.12	0.043	3.38, 20.42
Fixed-effect regression	8.18	2.75	0.003	2.77, 13.58
Mixed-effect regression	10.18	5.08	0.045	0.23, 20.13

Note: The coefficient captures the effect of the interaction (fattyacid^×^pten) on soma size.

Interaction effects are typically visualized in a manner similar to the plot in Fig 2C. The areas of interest on the graph are the differences in soma size between the vehicle control and fatty acid delivery in the control and Pten shRNA groups. If those differences are significant, then there is an interaction effect between Pten knockdown and fatty acid delivery. As shown in the plot, the increase in soma size in the fatty acid exposure treatment group (blue) is significantly more than the vehicle control group (red). The black dotted line demonstrates what the data would have looked like if there were no interaction between Pten knockdown and fatty acid exposure and the observed mean soma size for Pten = 0 was the same as in the current study. The difference between the dotted black line and the blue solid line, denoted by the curly bracket, for the group with Pten knockdown (Pten knockdown status = 1) is equal to the interaction effect.

## Discussion

**Fig 3:**
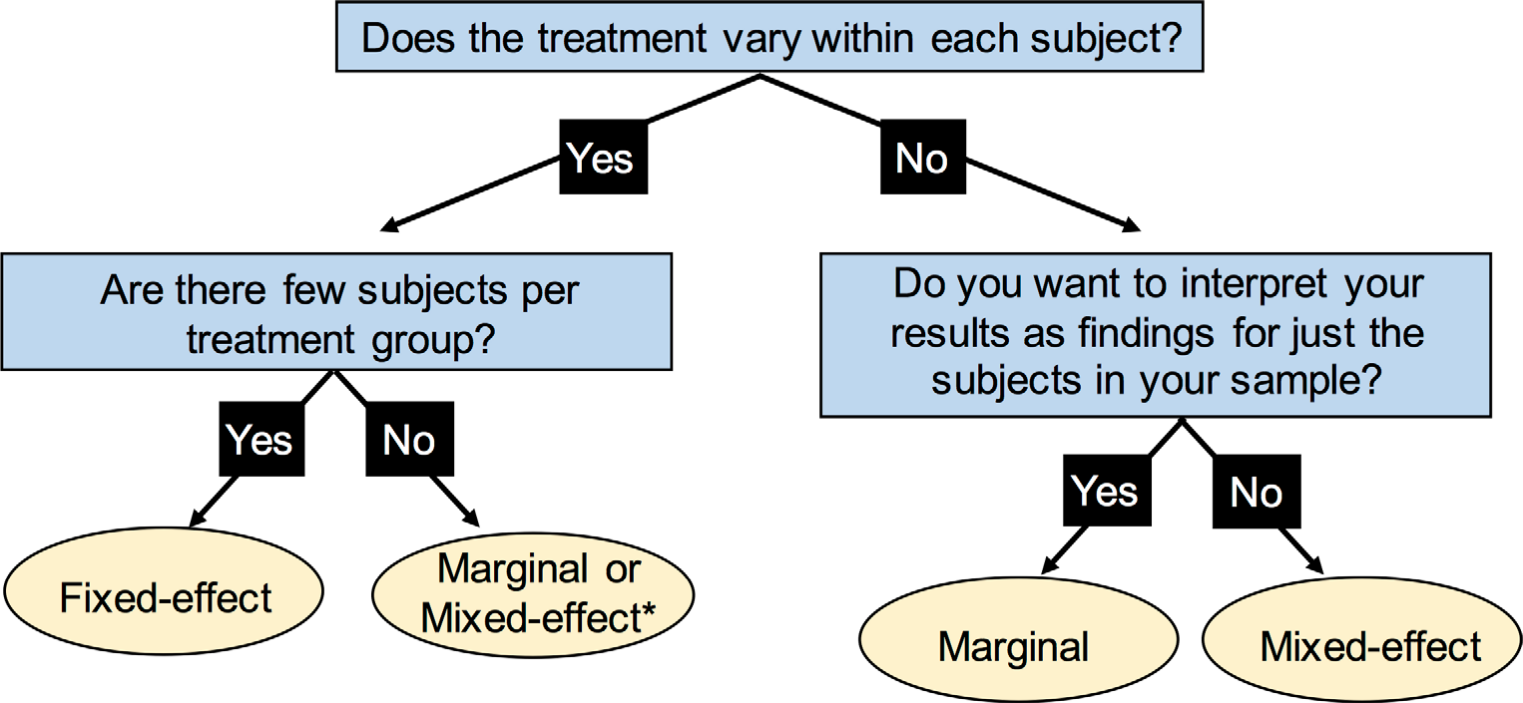
Main decision points for statistical analysis of clustered data. The flow chart outlines the primary questions researchers should address when weighing options of statistical research design of a study with clustered data. *Readers should refer to Table 2 for subtler differences between the marginal and mixed-effect model.

In this work, we have discussed several approaches for modeling clustered data generated from measuring multiple neurons per mouse. A naïve user of statistical software is likely to treat every neuron as an independent observation or, if they are more familiar with mouse studies in which one observation is obtained per mouse, to aggregate the data so that it conforms to this format. As described above, these naïve approaches are in general problematic and each preferred approach we have outlined has relative pros and cons. The major decision points researchers will come across when deciding which statistical approach is most appropriate for their study are outlined in the flow diagram (Fig 3), and Table 2 contains additional, more nuanced differences between the models described in this text.

The experimental data studied here had a high number of neurons per mouse, with relatively few mice per treatment group. Consequently, the results from the between-mouse effect showed considerable variability between approaches in the estimate of the effect size and the associated standard errors, which demonstrates how having few subjects per treatment group decreases consistency of the estimate across different models. In a study with clustered data, there are two distinct approaches to increasing sample size: increasing the number of subjects or the number of observations per subject. These approaches are not equivalent in their effects on the precision of the models. Having a larger number of subjects will increase the precision of the estimate for marginal and mixed-effect models but may make the fixed-effect model more cumbersome. The fixed-effect model, which works best for studies with a smaller number of subjects with a larger number of observations each, would instead benefit from increasing the number of measurements per subject. The marginal or mixed-effect model will not improve significantly in the precision of the estimate by adding more observations per subject because information about the variation between mice or the population mean is not being gained as much as it would be if more subjects were added to the study. Inclusion of the aggregate Pten predictor improved the stability of estimates across models, and therefore may be a beneficial approach for studies that have few subjects. The results from the within-mouse treatment effect were stable across the different approaches because there were numerous observations per mouse, and therefore the decision between the approaches matters less when analyzing a within-mouse treatment effect.

In general, mixed-effect models are especially suited for studies with a larger number of subjects to allow for more precise estimation of the variance between subjects, because the variance of the random effect distribution is an unknown parameter informed by variability between subjects [16]. The mixed-effect model can be improved by including the aggregate measure as a fixed effect (e.g. $\bar{\text{Pten}}$) if the proportion of observations with the treatment helps legitimize the assumption that the subject random effects are independent. While a precise recommendation on the number of subjects at which the mixed-effect model becomes viable depends on the amount of variability between subjects, if there are 5 or fewer subjects this model is not recommended.

A marginal regression model is best if one does not want to make assumptions about the distribution of the observations, or the researchers want to interpret their results as population-averaged instead of subject-specific effects [17].

Fixed-effect models can only be used when the intervention is at the individual-level. They are not reliant on the distribution of observations, but if there are only few observations per subject, the subject-level effects may be noisy. Fixed-effect approaches can be cumbersome if there are a large number of subjects, so they are best for studies with smaller numbers of subjects having similar and ideally many observations on each subject [18]. If a fixed effect model is used, it is important that the investigators provide the cautionary note that the results only pertain to the actual subjects in the study because the fact that the subjects are themselves a sample is ignored.

Generating consensus on specific guidelines for how to choose the most appropriate approach among the models that handle clustered data is difficult, because not every study is cut and dry in terms of which method is best. While mixed-effect models are appealing, they do involve more assumptions than the marginal regression approach and on some occasions, such as studies with a within-mouse effect but a small number of mice, marginal or fixed-effect regression may be more appropriate. For this reason, we have provided suggestions on when to use each approach and how to implement it. We also strongly recommend that papers or their supplemental appendices contain sufficient details for others to reproduce the results. Clear guidelines and expectations are a good start for improving the quality of statistics and methods descriptions in published studies [19], but it is still important to ensure the statistics being reported are valid for the study design.

Another important implication of clustered data is the influence it has on effective sample-sizes of experiments. Like all scientific experiments involving mouse models, those in neuroscience confront the competing issues of time and cost with the ethical considerations of using enough mice to get sufficient statistical power to detect an effect of clinical significance. In order to conduct humane research, there are several guidelines in place encouraging study designs that reduce the number of mice needed [20]. It is acknowledged that taking repeated measures is an approach that can be used to increase power without increasing sample-sizes, but any correlation within subjects must be accounted for in statistical analyses [20].

In conclusion, we recommend utilizing regression models that account for clustering, such as marginal, fixed-effect, or mixed-effect models, when analyzing data that have multiple measurements per subject. Distinguishing between these models should be based on the criteria listed in Table 2. Performing appropriate statistical tests and using the corresponding statistical inferential statements should be a high priority to all authors hoping to publish their work, and proper data analysis will help ensure the reproducibility and validity of novel scientific findings.

## Supporting Information

S1 Appendix Calculation of effective sample size with ICC. (DOCX)
