# Supplementary material for: Analyzing Clustered Data: Why and How to Account for Multiple Observations Nested within a Study Participant?
Source: PLoS One. 2016 Jan 14;11(1):e0146721. doi: 10.1371/journal.pone.0146721 (PMC4713068; doi:10.1371/journal.pone.0146721)
Supplement: S1 Appendix — (DOCX) [file pone.0146721.s001.docx]

S1 Appendix

**Calculation of effective sample size with ICC**

An important consequence of clustered data is the influence clustering has on the sample-size needed to determine a given level of statistical power for a study. The calculation requires some prior information regarding the intraclass correlation coefficient (ICC), which could be obtained from a pilot study, for example. The ICC compares the variance between clusters ($\tau^{2}$) to the variance within clusters ($\sigma^{2}$), and is calculated with the following equation:

$ICC= \tau^{2}/(\tau^{2}+ \sigma^{2})$ (1)

The ICC can be used to calculate the “design effect” (DE), or the estimate of the extent to which sample-size should be altered due to clustering of data:

$DE=1+\left( n-1 \right)*ICC$ (2)

where n equals the average cluster size. Once the DE is known, it can be used to approximate the “effective sample-size” (ESS) of the study (N):

$N=(n*k)/DE$ (3)

where k = the number of clusters. Prior knowledge about the ICC can be helpful in predicting how much accounting for the between-mouse variation in the statistical model will affect the results.

The ICC can be calculated with statistical software such as Stata following estimation of a mixed-effect model. In this experiment, the ICC of neurons within a mouse equals 0.175. The number of clusters, or mice exposed to either vehicle control or fatty acid (k) is 9 and the average number of neurons per mouse (n) equals 32.8. Therefore, using Equations 2 and 3 we can calculate the effective sample-size of the neuroscience study analyzed in the main text to be 123:

$$DE=1+\left( 9-1 \right)*0.175=2.4$$

$$N=\frac{32.8*9}{2.4}=123$$

This is reduced from the total number of observations, 295. However, it is still much higher than the number of mice in the study, demonstrating that reducing the data to a mean measurement per mouse is too harsh of a penalization to the power of the study. In a power calculation for a future study that is similar to the current study, one could use DE to appropriately scale the sample-size and then use the ESS (N) as the sample-size in formulae for a study with independent observations.
